# Identification and role of differentially expressed genes/proteins between pulmonary tuberculosis patients and controls across lung tissues and blood samples

**DOI:** 10.1002/iid3.1350

**Published:** 2024-07-18

**Authors:** Qifeng Li, Kuerbanjiang Maierheba

**Affiliations:** ^1^ Xinjiang Hospital of Beijing Children's Hospital Children's Hospital of Xinjiang Uygur Autonomous Region, Xinjiang Institute of Pediatrics Urumqi China; ^2^ Department of Nutrition and Food Hygiene, College of Public Health Xinjiang Medical University Urumqi China

**Keywords:** differentially expressed genes, differentially expressed proteins, proteomics, transcriptomics, tuberculosis

## Abstract

**Background:**

Differentially expressed genes/proteins (DEGs/DEPs) play critical roles in pulmonary tuberculosis (PTB) diagnosis and treatment. However, there is a scarcity of reports on DEGs/DEPs in lung tissues and blood samples in PTB patients.

**Objective:**

We aim to identify the DEGs/DEPs in lung tissues and blood samples of PTB patients and investigate their roles in PTB.

**Materials and Methods:**

The lung granulomas and normal tissues were collected from PTB patients for proteomic and transcriptomic analyses. Gene Ontology (GO) and Kyoto Encyclopedia of Genes and Genomes (KEGG) analyses annotated the functions of DEGs/DEPs. The GSE107994 data set was downloaded to identify the DEGs/DEPs in peripheral blood. The common DEGs and DEPs were identified. A nomogram was established. Pearson correlation analysis was conducted.

**Results:**

Eighty‐three DEGs/DEPs were identified. These DEGs/DEPs were mainly enriched in the movement of cell or subcellular components, regulation of cellular component biogenesis, and actin filament‐based process as well as in the pathways of inositol phosphate metabolism, adherens junction, phosphatidylinositol signaling system, leukocyte transendothelial migration, regulation of actin cytoskeleton, and tight junction. There were eight common DEGs/DEPs (TYMP, LAP3, ADGRL2, SIL1, LMO7, SULF 1, ANXA3, and PACSIN3) between the lung tissues and blood samples. They were effective in predicting tuberculosis. Moreover, the activated dendritic cells, macrophages, monocytes, neutrophils, and regulatory T cells were significantly positively correlated with TYMP (*r* > .50), LAP3 (*r* > .50), SIL1 (*r* > .50), ANXA3 (*r* > .5), and PACSIN3 (*r* < .50), while negatively correlated with LMO7 (*r* < −0.50) (*p* < .05). ADGRL2 and SULF1 did not have a significant correlation (*p* > .05).

**Limitations:**

The sample size was small.

**Conclusions:**

Eight common DEGs/DEPs of lung tissues and blood samples were identified. They were correlated with immune cells and demonstrated predictive value for PTB. Our data may facilitate the diagnosis and treatment of PTB.

## Graphical abstract

Eight common differentially expressed genes/proteins(DEGs/DEPs) of lung tissues and blood samples were identified, including TYMP, LAP3, ADGRL2, SIL1, LMO7, SULF1, ANXA3 and PACSIN3. They may be associated with the inflammation of tuberculosis patients.

## 1 INTRODUCTION

Tuberculosis is a chronic infectious disease caused by *Mycobacterium tuberculosis* (*M. tuberculosis*) and has become a public health issue that seriously affects human health.^1^, ^2^ According to the Global Tuberculosis Report published by the World Health Organization, the number of new tuberculosis cases worldwide declined from 7.1 million in 2019 to 5.8 million in 2020.^3^ However, the number increased to 6.4 million in 2021.^3^ Tuberculosis remains a persistent issue in China, representing 7.4% of all estimated incident cases worldwide in 2021.^3^ *M. tuberculosis* can infect various organs and tissues throughout the body, including the intestines, lungs, joints, lymph nodes, spine, and genitourinary system. Of these, pulmonary tuberculosis (PTB) is the most common.^4^ Following infection, *M. tuberculosis* is recognized and phagocytosed by macrophages, and the antigenic information of *M. tuberculosis* is presented to T cells, thereby activating the adaptive immunity of the body.^5^ Macrophages can also recruit neutrophils, monocytes, T lymphocytes, and B lymphocytes by producing cytokines and chemokines, which can eliminate *M. tuberculosis* and play an important role in the occurrence and development of tuberculosis.^1^, ^6^

The differentially expressed genes (DEGs)/differentially expressed proteins (DEPs) may serve as targets for the diagnosis and treatment of tuberculosis.^7^, ^8^, ^9^, ^10^, ^11^ Feng et al. found that cholesterol‐induced leucine aminopeptidase 3 (LAP3) could serve as a potential novel candidate biomarker for the diagnosis of nonalcoholic fatty liver disease.^12^ However, its role in PTB remains undetermined. Another study showed that anti‐inflammatory‐related genes like ANXA1 (Annexin A1), GILZ (glucocorticoid‐induced leucine zipper), NFKBIA (NF‐κB inhibitor alpha), and NFKBIB (NF‐κB inhibitor beta) in the peripheral blood monocytes of patients with PTB were significantly upregulated.^10^ Recently, Li et al. reported that a 5‐gene model consisting of immune‐related genes, namely thymidine phosphorylase (TYMP), C5, GRN, IL1B, and IL23A, exhibited a strong diagnostic potential for tuberculosis.^11^ Several studies have demonstrated that the assessment of changes in blood transcript abundance on a genome‐wide scale between healthy and diseased states can offer a comprehensive understanding of the impact of *M. tuberculosis* infection on host defense.^13^, ^14^ This approach also provides a reliable method for identifying novel biomarkers for tuberculosis. However, the DEGs/DEPs in the lung tissues and the blood samples of patients with PTB, compared to healthy controls, have been rarely reported.

Herein, we analyzed the key DEGs/DEPs in the lung tissues and the blood samples of patients with PTB, aiming to facilitate the diagnosis and treatment of PTB. Our findings may also enhance the understanding of the immune and pathogenic mechanisms involved in the development of PTB.

## 2 MATERIALS AND METHODS

### 2.1 Study design

This is a retrospective experimental study, which was conducted from September 2020 to March 2022.

### 2.2 Study participants

We enrolled the participants from November 2020 to September 2021. In detail, three patients with PTB, who required surgery to remove nonfunctional lung granulomas, were recruited for lung tissue collection. The granuloma tissues from the lesion area and the normal lung tissues were collected from each patient. Another 35 patients with PTB and 22 healthy controls who underwent physical examination during the same time were recruited for blood sample collection. The baseline clinical data of study participants are presented in Table 1. Fresh peripheral blood samples were collected and plasma samples were obtained after centrifugation. This study was approved by the Ethics Committee of the Children's Hospital of Xinjiang Uygur Autonomous Region (Approval No. KY2022031226) and all methods were also performed following the relevant guidelines and regulations under the committee supervision. Informed consent was obtained from each patient.

**Table 1:** Baseline characteristics of participants.

Groups	Healthy control (*n* = 22)	Patients with pulmonary tuberculosis for blood sample collection (*n* = 35)	Patients with pulmonary tuberculosis for lung sample collection (*n* = 3)
Age, years	31 ± 3.6	33 ± 4.8	30 ± 2.7
Gender			
Male	12 (54.5%)	19 (54.3%)	2 (66.7%)
Female	10 (45.5%)	16 (45.7%)	1 (33.3%)
Sputum smear‐positive	0 (0)	11 (31.4%)	2 (66.7%)
Sputum culture positive	0 (0)	10 (28.6%)	2 (66.7%)
T‐SPOT.TB	13 (59.1%)	20 (57.1%)	2 (66.7%)
History of tuberculosis exposure	0 (0)	17 (48.6%)	3 (100%)
HIV+	0 (0)	0 (0)	0 (0)
Clinical symptoms			
Cough	0 (0)	35 (100%)	3 (100%)
Fever	0 (0)	33 (94.3%)	3 (100%)
Night sweat	0 (0)	28 (80.0%)	2 (66.7%)
Lassitude	0 (0)	20 (57.1%)	3 (100%)
Weight loss	0 (0)	27 (77.1%)	3 (100%)

Abbreviations: HIV, human immunodeficiency virus; T‐SPOT.TB, T cell spot test for tuberculosis infection.

### 2.3 Inclusion and exclusion criteria

All participants were aged between 18 and 59 years. Inclusion criteria for patients with PTB were as follows: (1) The diagnosis of PTB followed the Diagnostic Criteria for Pulmonary Tuberculosis in China (WS288–2008)^15^; (2) patients who did not receive antituberculosis drugs or antibiotics before enrollment; and (3) patients with a history of tuberculosis exposure. The exclusion criteria for PTB patients included (1) patients with comorbidities such as diabetes mellitus, autoimmune diseases, pulmonary tumors, or other pulmonary infections; (2) patients who had received immunomodulatory agents; and (3) patients who were aged over 60 years old. The healthy control group consisted of individuals with no bacteriological or clinical signs of PTB, which was confirmed through negative sputum smears or cultures.

### 2.4 Proteomic and transcriptomic analysis of the lung tissues

Proteomic and transcriptomic sequencing of lung tissues was conducted by Nohe Zhiyuan Biological Information Technology Co., LTD. For transcriptome sequencing, RNA samples were collected and sent for sequencing. The sequencing library was prepared after the removal of rRNA following the Illumina TruSeq RNA sample preparation guide (Illumina). The index adaptor was ligated after synthesizing the double‐stranded cDNA. After size selection with Agilent 2100 bioanalyzer, samples were submitted to Illumina PE150 (Illumina) for pair‐end sequencing of 2 × 150 bp. Raw reads were filtered using SOAPnuke (version 1.0.1) and then mapped to the human (hg19) genomes provided by Illumina iGenomes (Download source: cufflinks. cbcb. umd. edu/igenomes.html) using Tophat2 (version 2.0.7) with default settings. Alignment and quantification gene expression analysis were performed using Stringtie. For proteomic sequencing, proteins were labeled by TMT® Mass Tagging Kits and Reagents (Thermo Fisher Scientific). Fraction separation, LC‐MS/MS analysis, and data analysis, including protein identification and quantification, were performed using proteome discoverer 2.4 (PD2.4; Thermo).

The “edgeR” software package was used to analyze the DEGs and DEPs between the granuloma tissues from the lesion area and the normal lung tissues. The volcano plot was generated based on fold change (FC) of protein/gene expression and *p*‐value using ggplot2 software. The heatmap was plotted with ggplot2. The screening criteria for differential expression were |log2FC | |>1 and *p‐v*alue < .05.^16^, ^17^ The common DEGs and DEPs in the lung tissues were obtained by using the online Venn Diagram software (http://bioinformatics.psb.ugent.be/webtools/Venn/).

### 2.5 Functional analysis

The latest gene annotation, which was obtained from the org.Hs.eg.db package (version 3.1.0), was used as a background. Then, DEGs/DEPs were mapped to the gene sets in the background. ClusterProfiler package (version 3.14.3) was used to perform Gene Ontology (GO) enrichment analysis and Kyoto Encyclopedia of Genes and Genomes (KEGG) pathway analysis of DEGs.^18^, ^19^, ^20^, ^21^ Functional analysis was conducted using the InterProScan program. The gene sets ranged from 5 to 5000, and *p* < .05 was considered to be statistically significant.

### 2.6 Transcriptomic analysis of the blood samples from the GEO public database

The GSE107994 data set was downloaded from the GEO public database and then normalized. The DEGs/DEPs of blood samples between healthy controls and patients with PTB were identified using the R package “edgeR.” The volcano plot was plotted with ggplot2 software based on the FC of gene expression and *p*‐value. The heatmap was plotted with ggplot2. The |log2FC | |>1 and *p*‐value < .05 were used to screen DEGs/DEPs. The common DEGs/DEPs between the lung tissues and the blood samples of patients with PTB were obtained by using online Venn diagram software (http://bioinformatics.psb.ugent.be/webtools/Venn/).

### 2.7 Nomogram prediction, receiver operating characteristic curve (ROC), and logistic regression analysis of common DEGs/DEPs

The R package pROC (version 1.17.0.1) was used for ROC analysis. The area under the curve (AUC) was calculated, which was used to indicate the accuracy of the common DEGs/DEPs in the diagnosis of patients with PTB.

The R software package rms were used to integrate the data of the common DEGs/DEPs, and the logistic method was used to establish a nomogram to evaluate the significance of the DEGs/DEPs in GSE107994 data, to compare the risk of tuberculosis caused by the DEGs/DEPs predicted by the model with the actual prevalence of tuberculosis. The multifactor regression model was constructed. Then, each DEG/DEP was assigned a score and the scores of DEGs/DEPs were summed to obtain total points according to the contribution of DEGs/DEPs to the prevalence of tuberculosis. The predictive value of DEGs/DEPs for tuberculosis was calculated by the function conversion relationship between the total points and the probability of outcomes.

### 2.8 Evaluation of peripheral blood immune cells of patients with PTB and healthy controls from the GEO public database

Based on the immune gene set in R package GSVA16, the immune characteristics of each GSE107994 sample were estimated by the ssGSEA algorithm. The ggplot2 software package was used to draw the boxplot of the proportion of immune cells.

### 2.9 Statistical analysis

The data, which presented as the mean ± SD, were analyzed using SPSS 22.0 statistical software. A *t*‐test was conducted to examine group differences. Pearson correlation analysis between the common DEGs/DEPs and peripheral blood immune cells was performed using the Hmisc package. The correlation coefficient was recorded. Statistical significance was defined as a *p* value < .05.

## 3 RESULTS

### 3.1 Analysis of DEPs in the lung tissues

**Figure 1:**
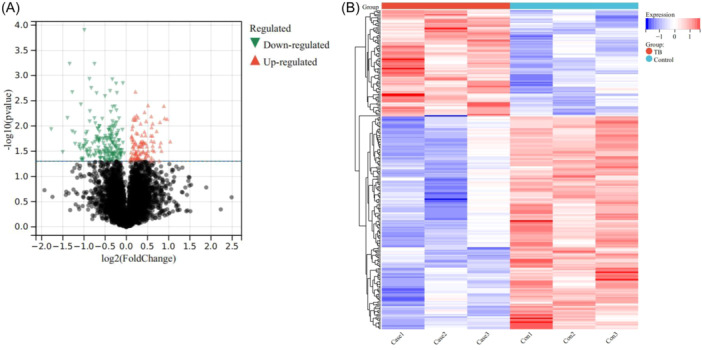
Analysis of DEPs in the lung tissues. Volcano plot (A) and heat map (B) showing differentially expressed proteins (DEPs) between the granuloma tissues from the lesion area and the normal lung tissues.

The granuloma tissues and normal tissues in the lungs of patients with PTB (*n* = 3) were collected for proteomic analysis. Volcano plot (Figure 1A) and heat map (Figure 1B) analysis showed that 249 DEPs were identified between granuloma tissues and normal tissues.

### 3.2 Analysis of DEGs in the lung tissues

**Figure 2:**
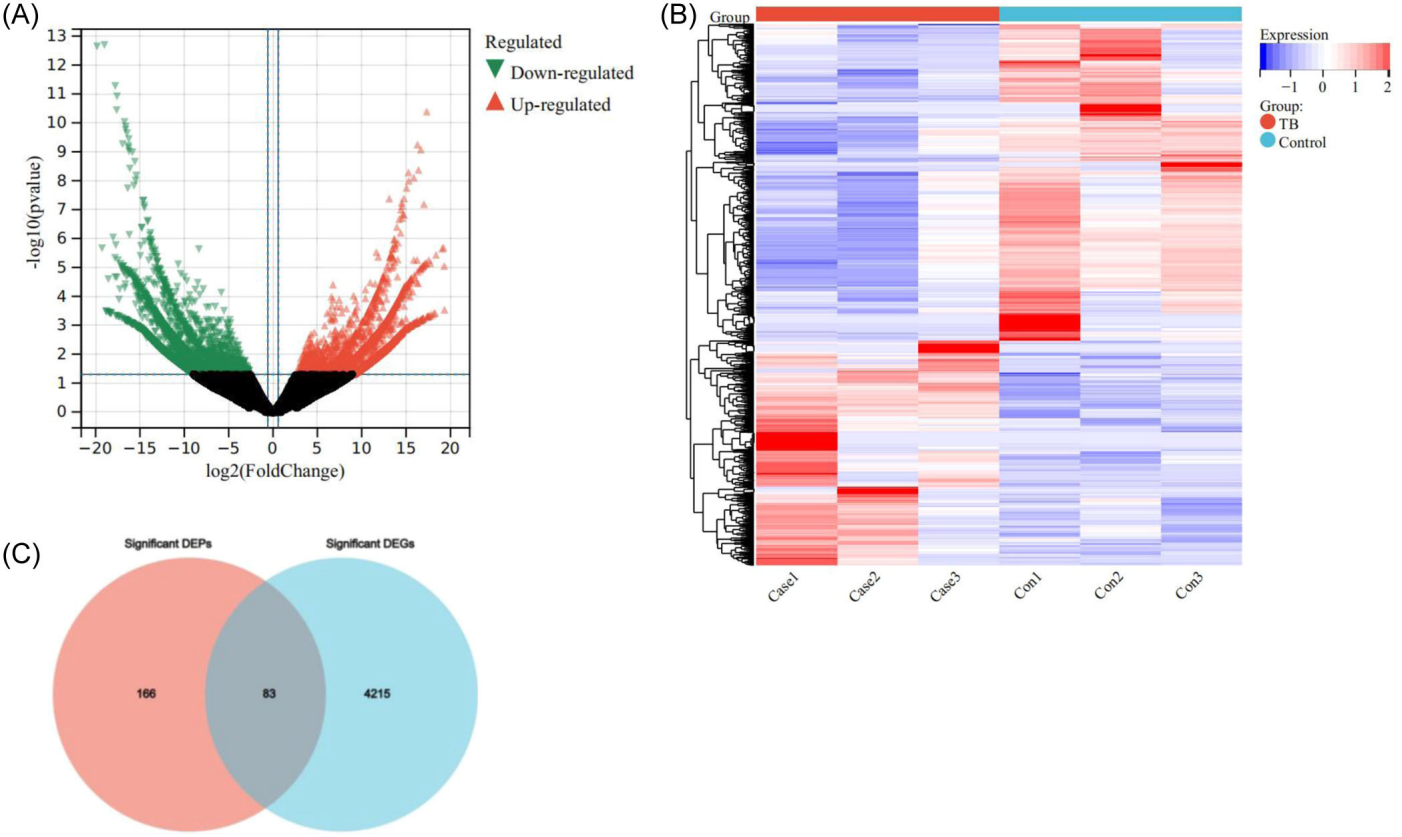
Analysis of DEGs in the lung tissues. Volcano plot (A) and heat map (B) showing differentially expressed genes (DEGs) between the granuloma tissues from the lesion area and the normal lung tissues. (C) Venn diagram of significantly differentially expressed proteins (DEPs) and significantly differentially expressed genes (DEGs).

Transcriptomic analysis was also conducted on the granuloma tissues and normal tissues from the lungs of patients with PTB (*n* = 3). The results, which were shown as a volcano plot (Figure 2A) and heat map (Figure 2B), found that there were 4298 DEGs between granuloma tissues and normal tissues across all participants.

To further identify the key genes/proteins involved in PTB, the DEGs were compared with the DEPs from the same samples. The Venn diagram (Figure 2C) showed that there were 83 common DEGs/DEPs in the granuloma tissues and normal tissues.

### 3.3 GO enrichment analysis and KEGG pathway analysis of the 83 common DEGs/DEPs

**Figure 3:**
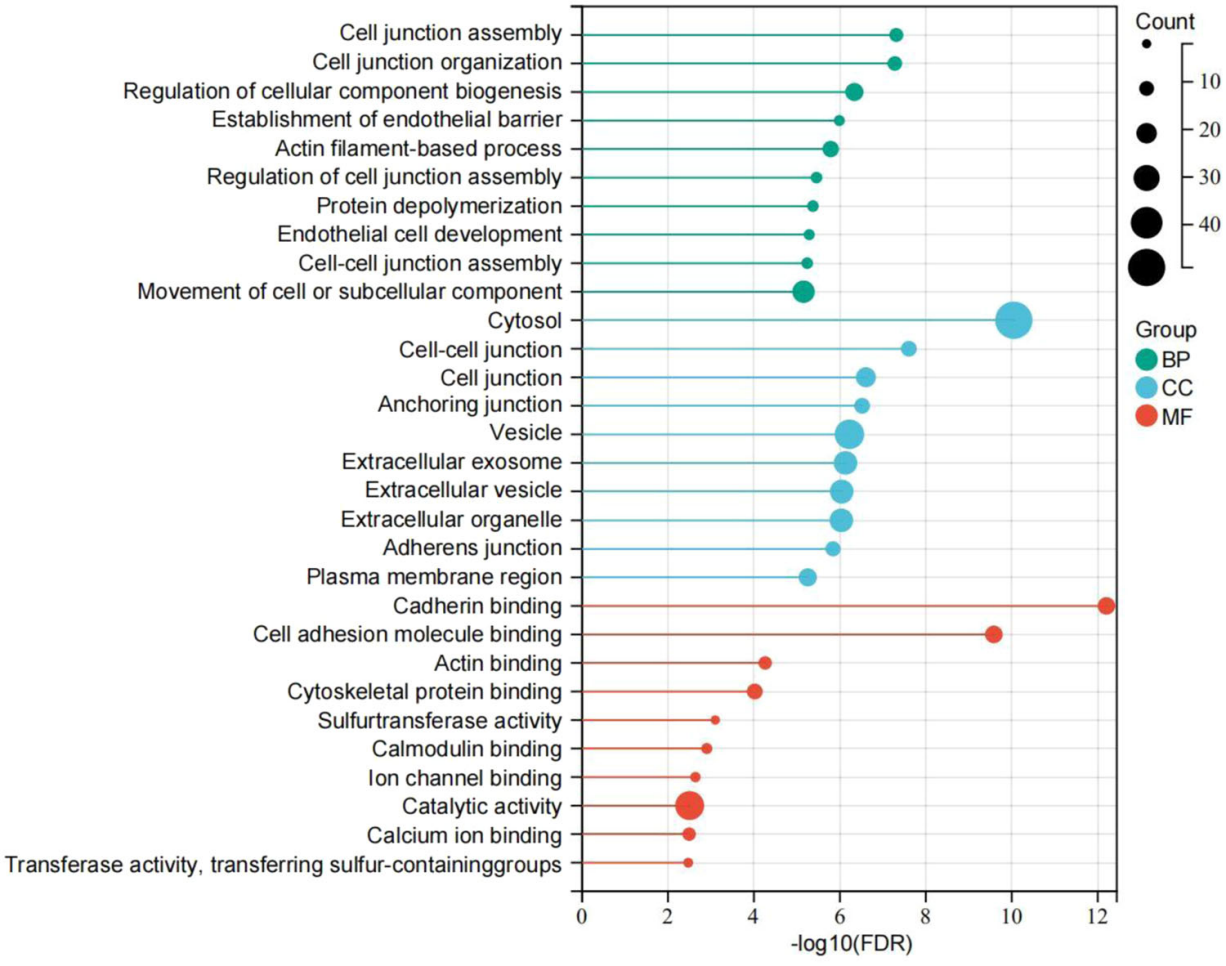
GO enrichment analysis. The top 10 enriched GO terms in biological process, cellular component, and molecular function are respectively shown. GO, Gene Ontology. BP, biological process; CC, cellular component; FDR, false discovery rate; MF, molecular function.

GO enrichment analysis, including cellular component, molecular function, and biological process, was performed on the 83 common DEGs/DEPs (Figure 3). For the cellular component, they were mainly enriched in the cytosol, vesicle, extracellular exosome, extracellular vesicle, and extracellular organelle. For the molecular function, they were mainly enriched in catalytic activity, cadherin binding, cell adhesion molecule binding, and cytoskeletal protein binding. For the biological process, they were mainly enriched in the movement of cell or subcellular components, regulation of cellular component biogenesis, and actin filament‐based process.

**Figure 4:**
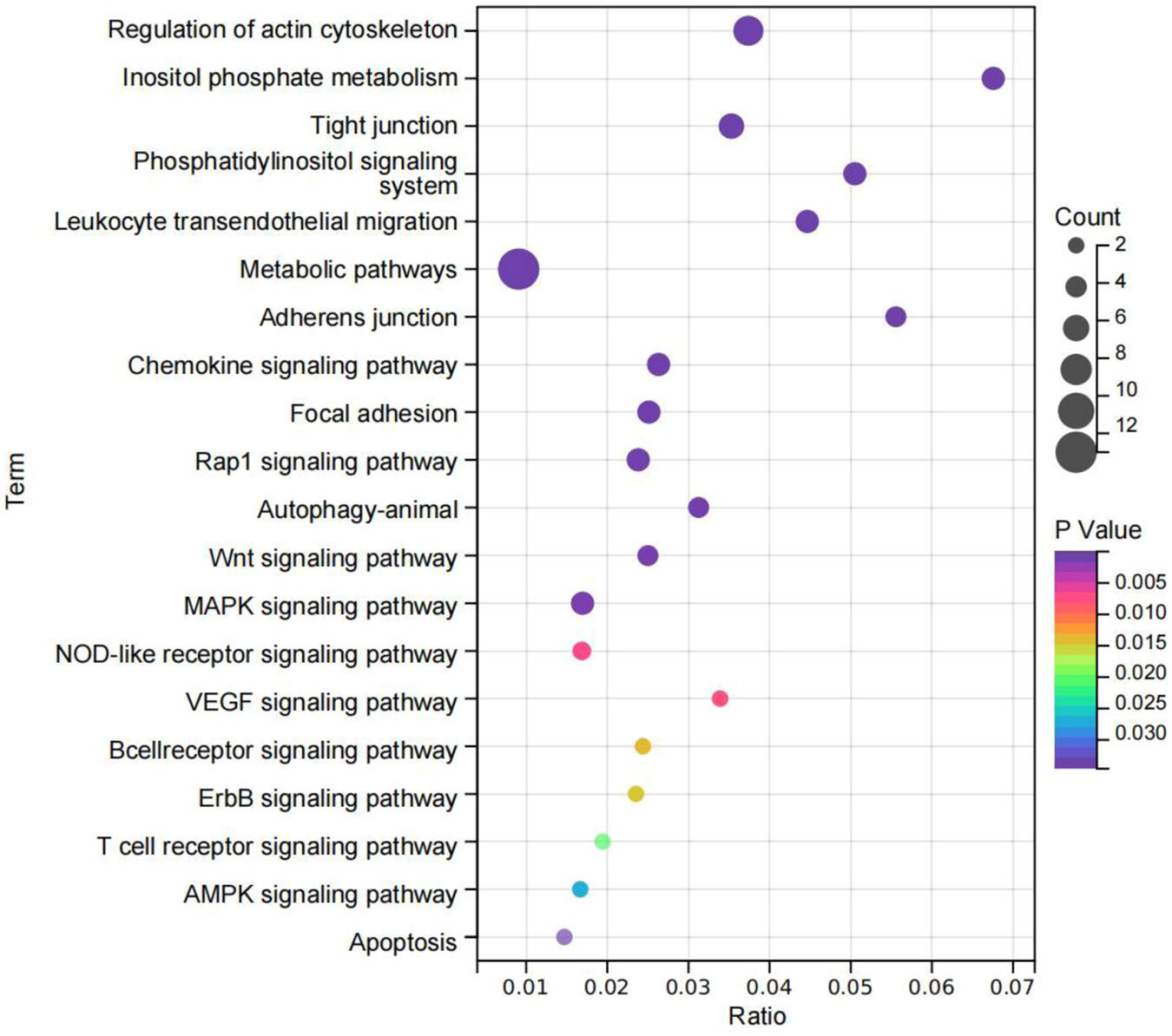
KEGG pathway analysis. The top 20 enriched KEGG pathways are listed. KEGG, Kyoto Encyclopedia of Genes and Genomes.

KEGG pathway analysis showed that the 83 common DEGs/DEPs were primarily enriched in the pathways of inositol phosphate metabolism, adherens junction, phosphatidylinositol signaling system, leukocyte transendothelial migration, regulation of actin cytoskeleton, and tight junction (Figure 4).

### 3.4 A total of eight common DEGs/DEPs between the peripheral blood and lung tissues are obtained

**Figure 5:**
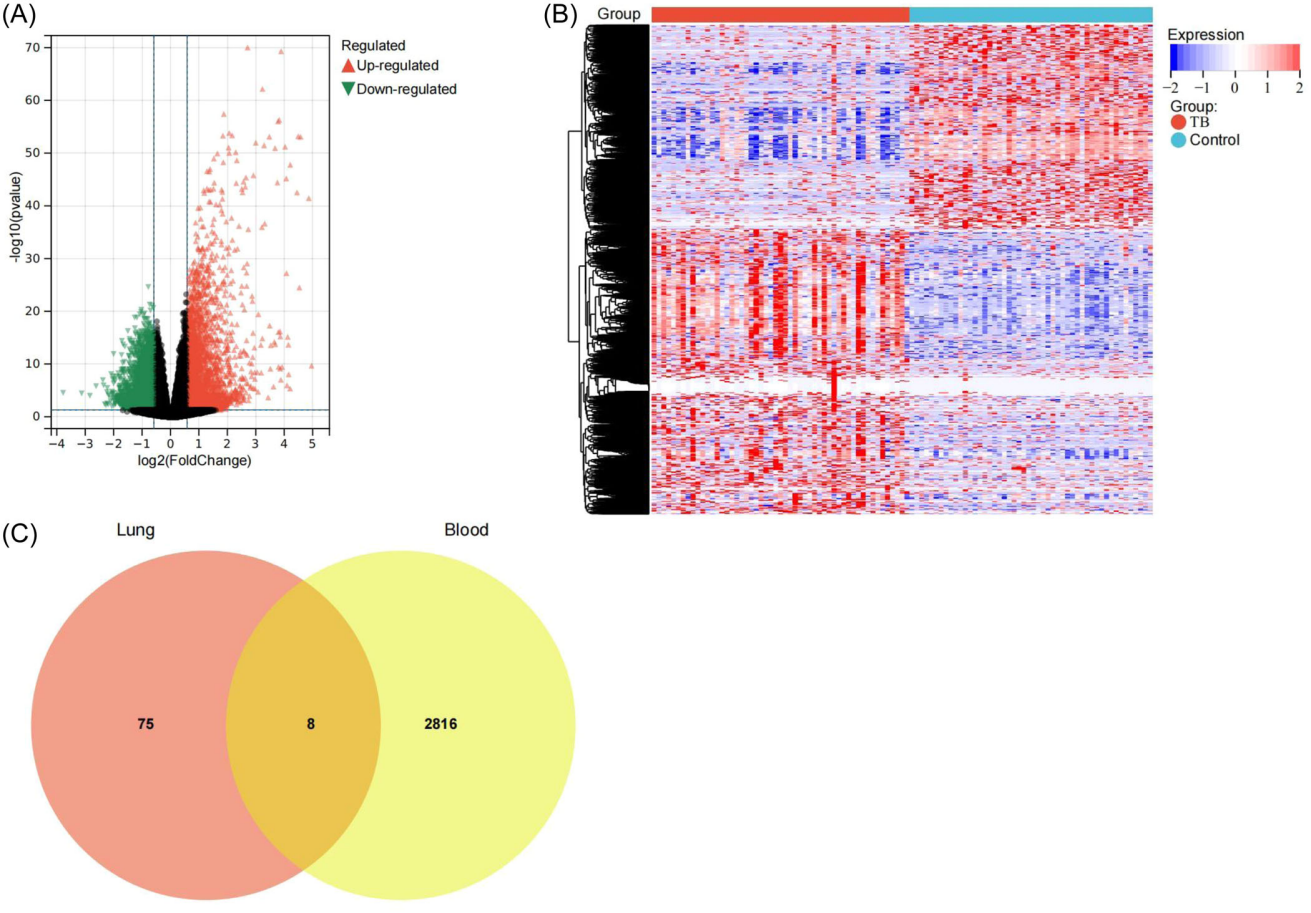
Analysis of DEGs in the peripheral blood of patients with pulmonary tuberculosis and healthy controls from the GSE107994 data set. Volcano plot (A) and heat map (B) displaying the differentially expressed genes (DEGs) in the blood of patients with pulmonary tuberculosis and healthy controls in the GSE107994 data set. (C) Venn diagram of DEGs in the lung and blood samples of patients with pulmonary tuberculosis.

DEGs/DEPs in the peripheral blood of patients with PTB and healthy controls from the GSE107994 data set and DEGs/DEPs in lung tissues were analyzed by edgeR software. A total of 2824 DEGs were identified in the blood of patients with PTB and healthy controls (Figures 5A,B). Venn diagram of DEGs/DEPs in lung tissues (*n* = 83) and those in the blood (*n* = 2824) showed eight common DEGs/DEPs (Figure 5C), including TYMP, LAP3, adhesion G protein‐coupled receptor L2 (ADGRL2), systemic RNA interference deficient‐1‐like (SIL1), LIM domain only 7 (LMO7), sulfatase 1 (SULF1), annexin A3 (ANXA3), and protein kinase C and casein kinase substrate in neuron 3 (PACSIN3).

In detail, the expression of LMO7 was downregulated in the lung tissues and blood samples of patients with PTB (Table 2). On the contrary, LAP3 and SULF1 were upregulated in the lung tissues and blood samples of patients with PTB. The other genes showed inconsistent expression trends in proteomics and transcriptomics of the lung tissues and blood samples.

**Table 2:** The eight common differentially expressed genes in the lung tissues and blood samples.

Gene name	Proteomics (lung tissues)	Transcriptomics (lung tissues)	Transcriptomics (blood samples)
LMO7	down	down	down
ANXA3	down	down	up
TYMP	up	down	up
LAP3	up	up	up
PACSIN3	–	down	up
ADGRL2	down	up	up
SIL1	up	down	up
SULF1	up	up	up

Abbreviations: ADGRL2, adhesion G protein‐coupled receptor L2; ANXA3, Annexin A3; LAP3, leucine aminopeptidase 3; LMO7, lim‐domain only 7; PACSIN3, protein kinase C and casein kinase substrate in neuron 3; SIL1, systemic RNA interference deficient‐1‐like; SULF1, sulfatase 1; TYMP, thymidine phosphorylase.

### 3.5 The value of the eight common DEGs/DEPs in predicting tuberculosis

**Figure 6:**
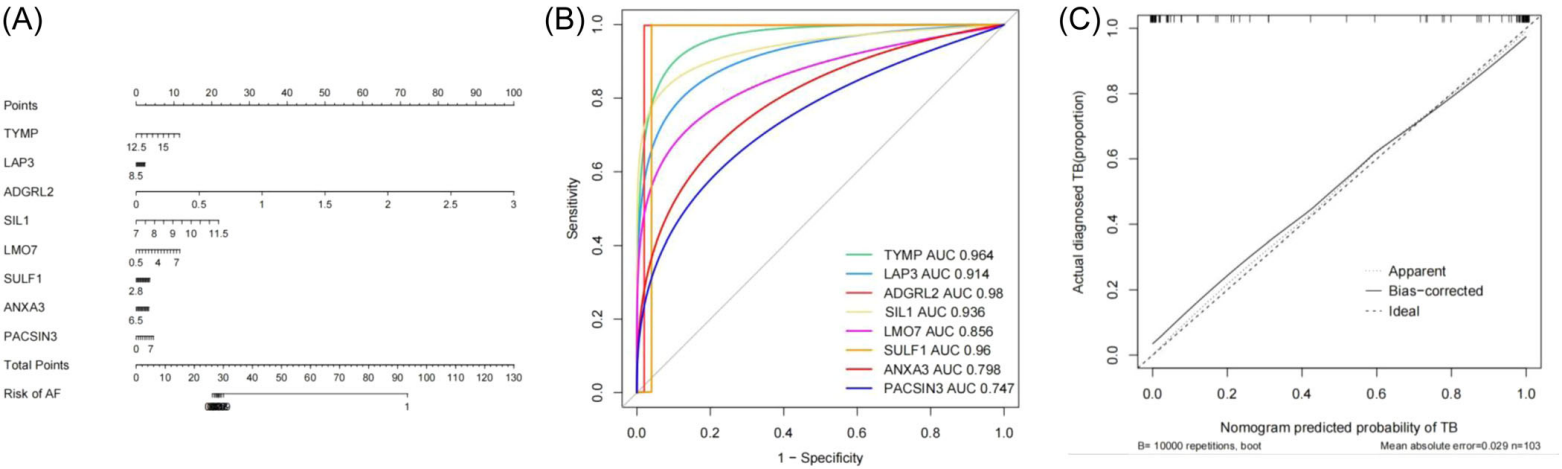
The value of the eight common differentially expressed genes (DEGs)/proteins (DEPs) in predicting tuberculosis. (A) The nomogram. The results of ROC analysis (B) and logistic regression analysis (C). ROC, receiver operating characteristic.

To predict the risk of tuberculosis, the nomogram was established based on the eight common DEGs/DEPs. The score of each DEG/DEP could be found by point scale in the nomogram (Figure 6A). ROC and logistic regression were used to assess the accuracy of the eight common DEGs/DEPs in predicting tuberculosis. The results of ROC showed that TYMP (AUC = 0.964), LAP3 (AUC = 0.914), ADGRL2 (AUC = 0.98), SIL1 (AUC = 0.936), LMO7 (AUC = 0.856), SULF1 (AUC = 0.96), ANXA3 (AUC = 0.798), and PACSIN3 (AUC = 0.747) had high accuracy in predicting tuberculosis (Figure 6B). Logistic regression analysis also showed that the eight common DEGs/DEPs were effective in predicting tuberculosis (Figure 6C).

### 3.6 Functional analysis of the eight common DEGs/DEPs

**Figure 7:**
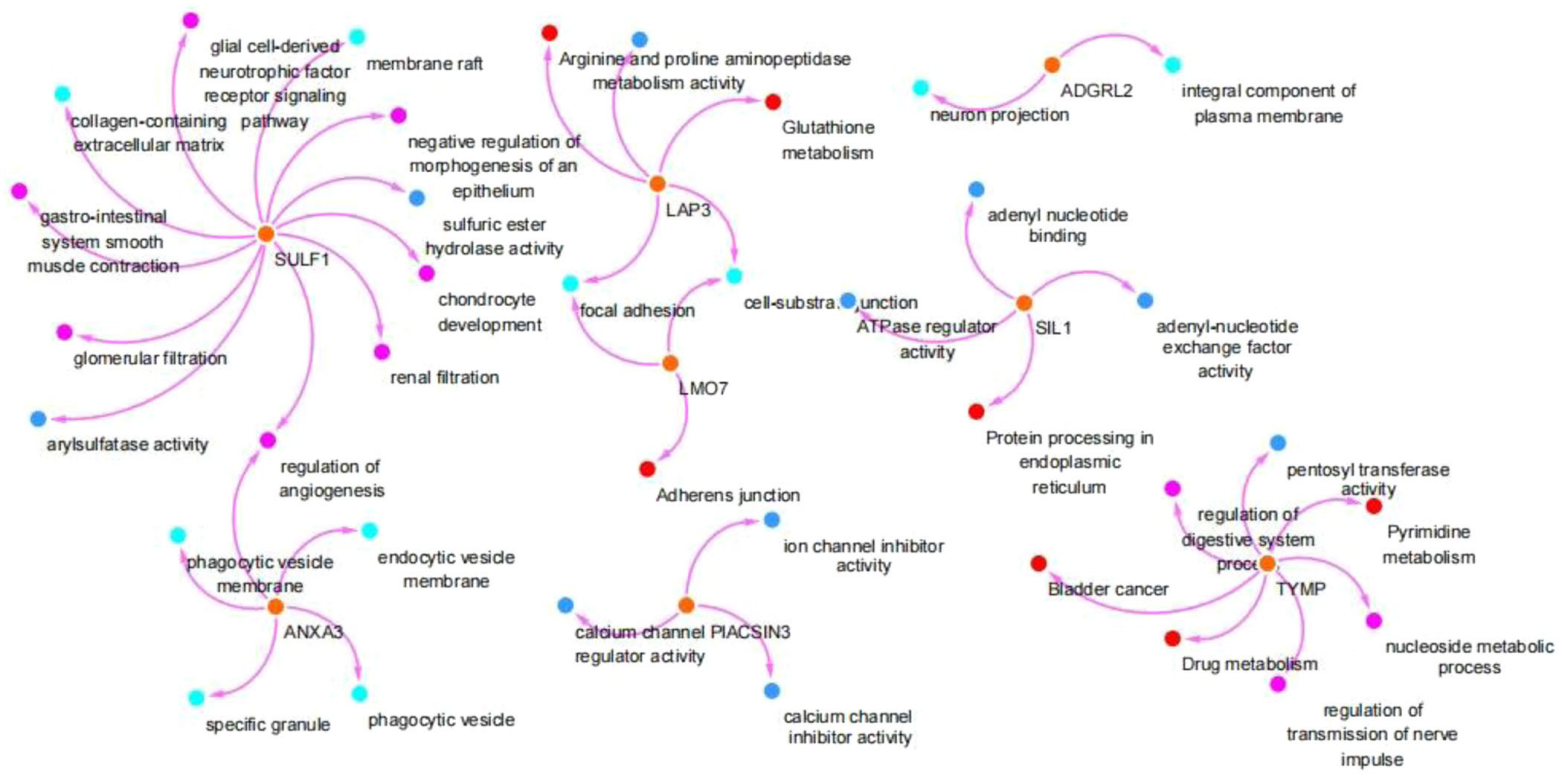
Functional analysis of the eight common differentially expressed genes (DEGs)/proteins (DEPs). The eight common DEGs/DEPs, including TYMP, LAP3, ADGRL2, SIL1, LMO7, SULF1, ANXA3, and, PACSIN3, and their biological functions are respectively shown.

Functional analysis showed that (Figure 7) SULF1 was associated with the regulation of angiogenesis, arylsulfatase activity, glomerular filtration, gastrointestinal system smooth muscle contraction, collagen‐containing extracellular matrix, glial cell‐derived neurotrophic factor receptor signaling pathway, membrane raft, negative regulation of morphogenesis of epithelium, sulfuric ester hydrolase activity, chondrocyte development, and, renal filtration. ANXA3 was associated with regulation of angiogenesis, endocytic vesicle membrane, phagocytic vesicle, specific granule, and, phagocytic vesicle membrane. LAP3 was associated with arginine and proline metabolism, aminopeptidase activity, glutathione metabolism, cell‐substrate junction, and focal adhesion. LMO7 was associated with focal adhesion, cell‐substrate junction, and adherens junction. PACSIN3 was associated with ion channel inhibitor activity, calcium channel inhibitor activity, and calcium channel regulator activity. ADGRL2 was associated with neuron projection and an integral component of the plasma membrane. SIL1 was associated with adenyl nucleotide binding, adenyl‐nucleotide exchange factor activity, protein processing in endoplasmic reticulum, and ATPase regulator activity. TYMP was associated with the regulation of the digestive system process, pentosyltransferase activity, pyrimidine metabolism, nucleoside metabolic process, regulation of transmission of nerve impulses, drug metabolism, and bladder cancer.

### 3.7 Assessment of peripheral blood immune cells of patients with PTB and healthy controls in the GSE107994 data set

**Figure 8:**
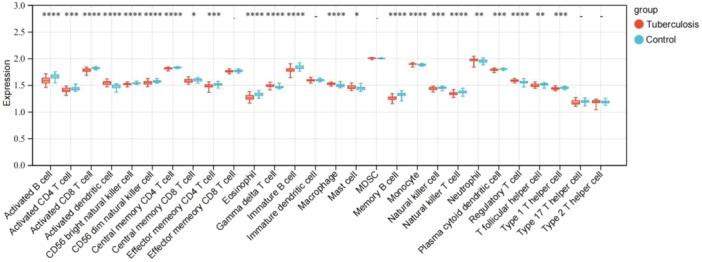
Boxplot of the proportion of immune cells of patients with pulmonary tuberculosis and healthy controls in the GSE107994 data set. As determined by the *t*‐test, the ****, ***, **, and * indicate significant differences of *p* < .0001, .01, .01, and .05. “‐” indicates no significant difference.

Comparison of peripheral blood immune cells by ssGSEA boxplot showed that the expression of activated dendritic cells, gamma delta T cells, macrophages, mast cells, monocytes, neutrophils, and regulatory T cells in the tuberculosis group were significantly higher than those in the control group (Figure 8). However, the expression of activated B cells, activated CD4 T cells, activated CD8 T cells, CD56bright natural killer cells, CD56dim natural killer cells, central memory CD4 T cells, central memory CD8 T cells, effector memory CD4 T cells, eosinophils, immature B cells, memory B cells, natural killer cells, natural killer T cells, plasmacytoid dendritic cells, T follicular helper cells, and type 1 T helper cells in the tuberculosis group were significantly lower than those in the control group.

### 3.8 Correlation analysis between the eight common DEGs/DEPs and peripheral blood immune cells

**Figure 9:**
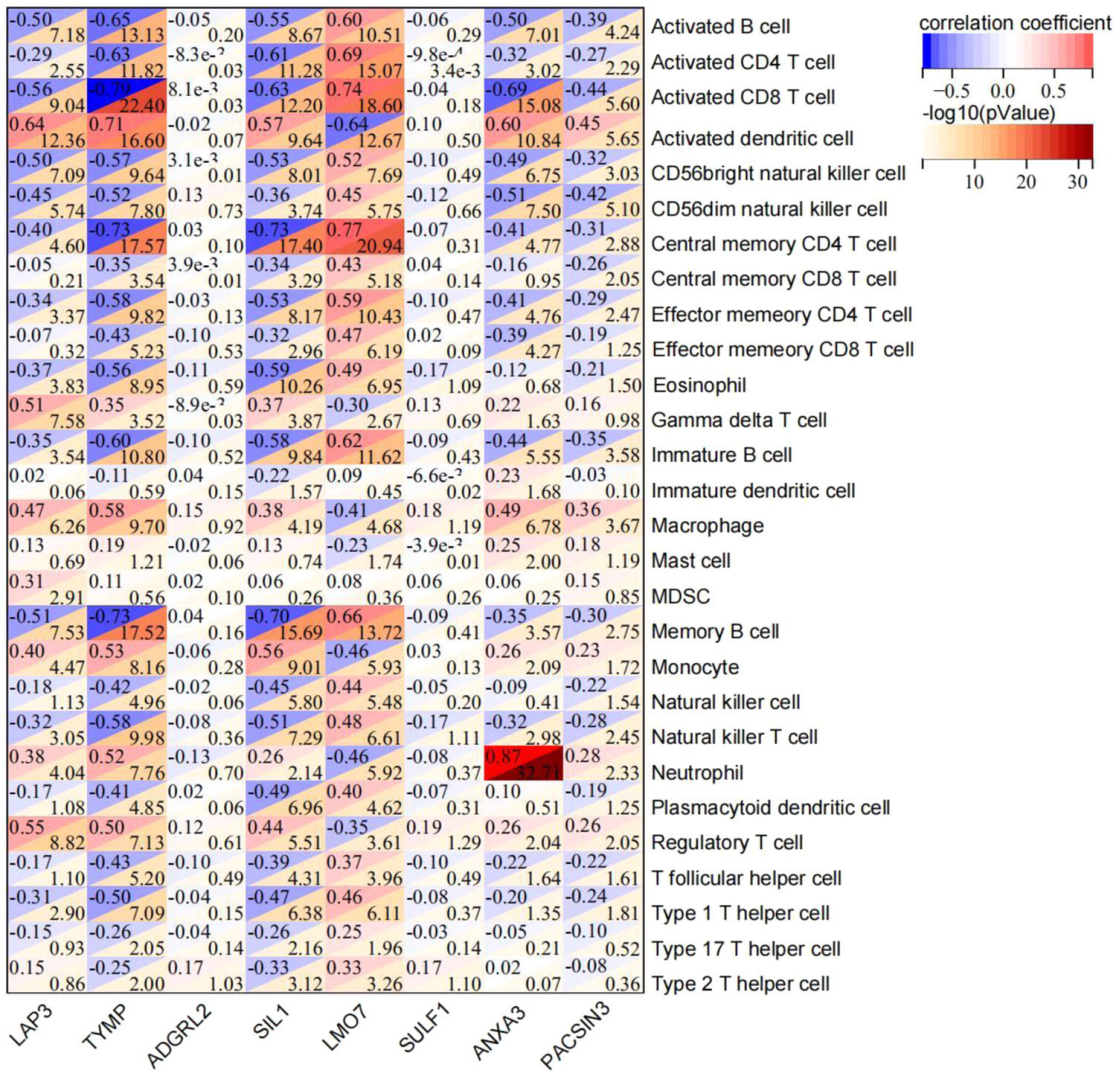
Pearson correlation analysis between the eight common differentially expressed genes (DEGs)/proteins (DEPs) and peripheral blood immune cells. The correlation of TYMP, LAP3, ADGRL2, SIL1, LMO7, SULF1, ANXA3, and PACSIN3 with the blood immune cells is shown. The red color shows a positive correlation and the blue color shows a negative correlation.

Pearson correlation analysis was performed to assess the correlation between the eight common DEGs/DEPs and peripheral blood immune cells (Figure 9). TYMP showed a strong negative correlation with activated B cells (*r* = −.65), activated CD4 T cell (*r* = −.63), activated CD8 T cell (*r* = −.79), CD56bright natural killer cell (*r* = −.57), CD56dim natural killer cell (*r* = −.52), central memory CD4 T cell (*r* = −.73), effector memory CD4 T cell (*r* = −.58), eosinophil (*r* = −.56), immature B cell (*r* = −.60), memory B cell (*r* = −.73), natural killer T cell (*r* = −.58), and, type 1 T helper cell (*r* = −.50). However, TYMP showed a strong positive correlation with activated dendritic cells (*r* = .71), macrophage (*r* = .58), monocyte (*r* = .53), neutrophil (*r* = .52), and regulatory T cell (*r* = .50). Moreover, LAP3 was strongly negatively correlated with activated B cells (*r* = −.50), activated CD8 T cell (*r* = −.56), CD56bright natural killer cell (*r* = −.50), and memory B cell (*r* = −.51), while it was strongly positively correlated with activated dendritic cell (*r* = .64), gamma delta T cell (*r* = .51), and regulatory T cell (*r* = .55). SIL1 had a strong negative correlation with activated B cells (*r* = −.55), activated CD4 T cell (*r* = −.61), activated CD8 T cell (*r* = −0.63), CD56bright natural killer cell (*r* = −.53), central memory CD4 T cell (*r* = −.73), effector memory CD4 T cell (*r* = −.53), eosinophil (*r* = −.59), immature B cell (*r* = −.58), memory B cell (*r* = −.70), and natural killer T cell (*r* = −.51), whereas it had a strong positive correlation with activated dendritic cell (*r* = .57) and monocyte (*r* = .56). There was a strong negative correlation of LMO7 with activated dendritic cells (*r* = −.64), and a strong positive correlation of LMO7 with activated B cells (*r* = .60), activated CD4 T cell (*r* = −.69), activated CD8 T cell (*r* = .74), CD56bright natural killer cell (*r* = .52), central memory CD4 T cell (*r* = .77), effector memory CD4 T cell (*r* = .59), immature B cell (*r* = .62), and memory B cell (*r* = .66). Additionally, a strong negative correlation was found between ANXA3 and activated B cells (*r* = −.50), activated CD8 T cells (*r* = −.69), and CD56dim natural killer cells (*r* = −.51). Meanwhile, a strong positive correlation was found between ANXA3 and activated dendritic cells (*r* = .60) and neutrophils (*r* = .87). The correlation between PACSIN3 and peripheral blood immune cells was not strong (*r* < .50). ADGRL2 and SULF1 did not have a significant correlation with peripheral blood immune cells (*p* > .05).

## 4 DISCUSSION

Tuberculosis is a chronic inflammation caused by *M. tuberculosis*. The immune cells, cytokines, and chemokines play important roles in tuberculosis. For example, the macrophages could participate in the recognition and phagocytosis of *M. tuberculosis*, and present the antigens to T cells, thereby initiating adaptive immunity.^5^ Macrophages could also produce cytokines, such as IL‐1β, IL‐6, and TNF‐α, to kill and eliminate pathogens.^1^, ^5^ Dendritic cells could also act as antigen‐presenting cells and present the antigens to T cells, thereby initiating adaptive immunity.^22^, ^23^ Furthermore, after stimulation by *M. tuberculosis*, macrophages could produce chemokines to recruit neutrophils, monocytes, T and B lymphocytes, and other immune cells to participate in the process of killing and eliminating pathogens.^1^ In addition, blood transcriptomics has unveiled the progression and resolution of the immune response in tuberculosis, thereby potentially facilitating the clinical management of the disease.^7^ In this study, the levels of macrophages, dendritic cells, monocytes, neutrophils, and regulatory T cells were significantly increased in the peripheral blood of patients with PTB, indicating that these immune cells may play an important role in tuberculosis.

Proteomic and transcriptomic analyses showed that 83 common DEGs/DEPs were identified in the lung tissues of PTB patients. Moreover, there were eight common DEGs/DEPs between lung tissues and blood samples of PTB patients, including TYMP, LAP3, ADGRL2, SIL1, LMO7, SULF1, ANXA3, and, PACSIN3. TYMP was expressed in macrophages, monocytes, and stromal cells.^24^, ^25^ Our study also showed a strong positive correlation between TYMP and macrophages/monocytes. TYMP expression was found to be upregulated in lung proteomics and blood transcriptomics in our study. TYMP, as a platelet‐derived endothelial‐cell growth factor, is considered to be involved in angiogenesis.^26^, ^27^, ^28^ Several studies showed that TYMP may play an important role in tumor development by promoting tumor angiogenesis.^26^, ^29^, ^30^, ^31^ Polena et al. found that the expression of vascular endothelial growth factor, which was involved in angiogenesis, was upregulated in the macrophages infected with *M. tuberculosis*.^32^ Some *Bacillus* species can survive within the granuloma and stimulate the granulomatous macrophages to secret angiogenic factors, thereby promoting angiogenesis, increasing vascular permeability, and leading to the escape of *M. tuberculosis* from the granuloma and the spread of *M. tuberculosis* throughout the body.^32^, ^33^, ^34^ Thus, granuloma formation during tuberculosis may be closely related to angiogenesis.

LAP3 is an extracellular peptidase that catalyzes the hydrolysis of amino‐terminal leucine residues of protein or peptide substrates.^35^ Studies have shown that LAP3 is also involved in the angiogenesis of various cancers,^36^, ^37^, ^38^ and has a potential role in regulating inflammation.^37^, ^38^, ^39^ A recent study showed that following SARS‐CoV‐2 infection, LAP3 was significantly upregulated along with inflammatory cytokines and chemokines.^39^ Therefore, the upregulated expression of LAP3 in the lung tissue and blood samples may be closely related to angiogenesis in the granulomas of patients with PTB and the level of inflammation induced by tuberculosis.

ANXA3, a calcium‐regulated phospholipid, and membrane‐binding protein, participates in membrane transport and the response of a series of calcium‐regulated proteins on the cell membrane surface, and plays an important role in the inflammatory response, cell growth and differentiation, and cytoskeletal protein response.^40^, ^41^ Studies showed that ANXA3 can bind to the phosphatidylserine of vesicles and mediate phagocytosis and granule degradation,^42^, ^43^ and mediate cell proliferation, apoptosis, and signal transduction of tumors.^44^ Consistently, our study also indicated that ANXA3 was associated with regulation of endocytic vesicle membrane, phagocytic vesicle, specific granule, and phagocytic vesicle membrane. ANXA3, which is considered an angiogenic factor, induces vascular endothelial growth factor production through the hypoxia‐inducible factor‐1 pathway and plays an important role in tumor metastasis by promoting angiogenesis.^45^, ^46^ The expression of ANXA3 was upregulated in the blood in our study. Similar to TYMP and LAP3, the upregulated expression of ANXA3 in the blood samples may be closely related to angiogenesis in the granulomas of patients with PTB.

PACSIN3 is mainly expressed in lung and muscle tissues and is involved in endocytosis.^47^, ^48^, ^49^ The current study showed that PACSIN3 was associated with ion channel inhibitor activity, calcium channel inhibitor activity, and calcium channel regulator activity. Studies have shown that transferrin endocytosis is blocked in a dose‐dependent manner in PACSIN3 overexpressing cells.^47^, ^49^ In this study, the expression of PACSIN3 was decreased in the lung tissues, which may contribute to the endocytosis of *M. tuberculosis* in the lung. However, the expression of PACSIN3 was increased in the blood of patients with PTB. The role of PACSIN3 should be further investigated.

LMO7 is a fibrous actin‐binding protein that is widely expressed at the apical surface of lung epithelial cells.^50^ The expression of the LMO7 gene was downregulated both in the lung tissues and blood samples of patients with PTB in this study. It is shown that LMO7 plays a role in the formation and maintenance of epithelial structures by remodeling the actin cytoskeleton.^51^ An animal study showed that LMO7‐deficient mice developed irregular and protruding epithelial lesions in the terminal and respiratory bronchioles at a young age, and these mice were susceptible to lung adenocarcinoma at an older age.^50^ Lung epithelial cells also play an important role in protecting against the infection of foreign microorganisms. They can provide physical and immunological barriers and stimulate an acquired immune response.^52^ In tuberculosis, lung epithelial cells can reduce the inflammatory response mediated by toll‐like receptors in macrophages.^53^ In this study, Pearson correlation analysis showed that LMO7 was negatively correlated with macrophages, suggesting a potential association between LMO7 levels and macrophages.

SULF1, which is a heparin‐degrading endosulfatase, can participate in and affect a variety of physiological and pathological processes by desulfurizing heparan sulfate proteoglycan on the surface of cells.^54^ Here, we found that the expression of SULF1 was upregulated in the lung tissues and blood samples of patients with PTB. The results of functional analysis indicated that the SULF1 was associated with the regulation of angiogenesis, and also involved in negative regulation of morphogenesis of an epithelium. Then the upregulated expression of SULF1 in the lung and blood samples may be closely related to angiogenesis in granulomas and pathological changes of lung epithelial cells induced by tuberculosis. Gong et al. reported that SULF1 had increased expression in colon cancer, and it was positively correlated with the infiltration of immune cells in colon cancer, including macrophages, dendritic cells, neutrophils, CD8+T cells, and CD4+T cells.^55^ Our results also showed that the levels of activated dendritic cells, macrophages, neutrophils, and regulatory T cells in the peripheral blood of patients with PTB were significantly increased, while Pearson correlation analysis did not show a significant positive correlation between SULF1 and immune cells. This study showed that SULF1 was related to the pathological changes of PTB, and had no significant relationship with the immune system of patients.

In addition, we also found that SIL1 was associated with adenyl nucleotide binding, adenyl‐nucleotide exchange factor activity, protein processing in the endoplasmic reticulum, and ATPase regulator activity. ADGRL2 was associated with neuron projection and integral components of the plasma membrane. However, these two genes are rarely reported. The role of these genes should be further investigated.

Notably, some of the identified common DEGs/DEPs were not specific to tuberculosis. As mentioned earlier, some identified DEGs/DEPs in our study, such as TYMP,^26^, ^27^, ^28^, ^29^, ^30^, ^31^ LAP3,^36^, ^37^, ^38^ ANXA,^44^, ^45^, ^46^ and SULF1,^55^ have also been implicated in angiogenesis and tumor development, as well as LAP3 showing increased levels post SARS‐CoV‐2 infection.^39^ This raises the important consideration of distinguishing among patients with tuberculosis, cancer, and other conditions with overlapping marker expression profiles. We suppose that for accurate patient distinguishment, a combination of markers and symptoms can be used. For example, a panel of markers, such as TYMP, LAP3, ANXA, and SULF1, along with traditional tuberculosis‐related symptoms such as persistent cough, weight loss, and fever, can be used to differentiate between patients with cancer, post‐SARS‐CoV‐2 infection, and those at risk for tuberculosis. Further studies are warranted for validation.

## 5 CONCLUSION

PTB remains a persistent issue and its underlying mechanisms and biomarkers remain to be further determined. Moreover, the DEGs/DEPs in the lung tissues and the blood samples of patients with PTB have been rarely reported. In this study, eight common DEGs/DEPs were identified, specifically TYMP, LAP3, ADGRL2, SIL1, LMO7, SULF1, ANXA3, and PACSIN3, from the lung tissues and blood samples of patients with PTB. These eight DEGs/DEPs play different roles and may be associated with the inflammation state and overall physiological functioning of patients with PTB. Additionally, they demonstrated predictive value for PTB. These DEGs are expected to facilitate the diagnosis and treatment of PTB. Our findings may also enhance the understanding of the immune and pathogenic mechanisms involved in the development of PTB. However, the specific functions and mechanisms of these DEGs/DEPs in PTB need to be further validated.

## AUTHOR CONTRIBUTIONS

Qifeng Li designed the study and performed the experiments. Kuerbanjiang Maierheba collected data, drafted the manuscript, and performed data analysis. Qifeng Li also contributed to manuscript writing. All authors read and approved the final manuscript.

## CONFLICT OF INTEREST STATEMENT

The authors declare no conflicts of interest.

## ETHICS STATEMENT

This study was approved by the Ethics Committee of Children's Hospital of Xinjiang Uygur Autonomous Region (Approval No. KY2022031226) and all methods were also performed following the relevant guidelines and regulations under the committee's supervision. Informed consent was obtained from each patient.

## Data Availability

The datasets generated and/or analyzed during the current study are available from the corresponding author upon reasonable request.
